# Central-type primitive neuroectodermal tumor of the uterus: Case report and literature review

**DOI:** 10.3389/fonc.2026.1926715

**Published:** 2026-10-23

**Authors:** Yi-Ying Lin, Ming-Yu Xie, Rong-Jun Mao, Bo-Tao Luo, Yan Qi

**Affiliations:** 1 Department of Pathology, Zhanjiang Central Hospital, Guangdong Medical University (Central People’s Hospital of Zhanjiang), Zhanjiang, Guangdong, China; 2 Department of Pathology and Research Center, Affiliated Hospital of Guangdong Medical University, Zhanjiang, Guangdong, China; 3 Department of Pathology, Foshan Hospital of Traditional Chinese Medicine, Guangdong, China

**Keywords:** central-type primitive neuroectodermal tumor (cPNET), differential diagnosis, histopathology, immunohistochemistry, small round cell tumor, uterus

## Abstract

Primitive neuroectodermal tumors (PNETs) arising in the uterus, particularly those classified as the central-type (cPNET), are exceedingly rare. To date, approximately 30 cases of uterine cPNET have been reported, and their clinicopathological features remain poorly characterized. These tumors typically present as highly aggressive small round cell neoplasms with variable neuroectodermal differentiation, often posing significant diagnostic challenges owing to their overlap with other uterine malignancies, including neuroendocrine carcinoma, endometrial stromal sarcoma, and undifferentiated carcinoma. Here, we report a rare case of uterine cPNET characterized by a diffuse small round cell morphology and prominent neuroendocrine differentiation. Immunohistochemical analysis demonstrated positivity for synaptophysin, INSM1, and CD56, whereas markers associated with Ewing’s sarcoma (CD99, FLI1 and NKX2.2) were negative. Next-generation sequencing revealed no Ewing sarcoma breakpoint region 1 rearrangements, supporting the exclusion of peripheral PNET. This case highlights the diagnostic complexity of uterine tumors with neuroectodermal differentiation and expands the current morphological and immunophenotypic spectra of this entity. Accurate recognition requires the integration of histopathology, immunoprofile, and molecular findings, with particular attention paid to the differential diagnosis of other high-grade uterine malignancies.

## Introduction

Primitive neuroectodermal tumors (PNETs) are a group of highly malignant tumors originating from primitive neuroepithelial cells that exhibit differentiation characteristics similar to those of neurons and glial cells in the central nervous system (CNS) (1). Traditionally, PNETs have been divided into peripheral-type (pPNET) and central-type (cPNET) tumors. pPNETs are closely related to Ewing’s sarcoma and are characterized by Ewing sarcoma breakpoint region 1 (EWSR1) rearrangements. In contrast, cPNET lacks EWSR1 fusion events and exhibits histological features of poorly differentiated tumors composed of small round blue cells, which may demonstrate varying degrees of neuroepithelial, glial, and ependymoid differentiation (2).

However, according to the World Health Organization (WHO) Classification of Female Genital Tumors (5th edition), the concept of PNET, particularly cPNET, has been increasingly reconsidered owing to its limited biological specificity. Tumors previously classified as cPNET are now thought to represent a heterogeneous group of neoplasms with neuroectodermal differentiation that overlaps with embryonal CNS tumors. In this context, diagnosis relies primarily on histomorphology and immunophenotype, with the exclusion of Ewing sarcoma (ES) through molecular testing, especially in the absence of EWSR1-related fusions (3). Emerging molecular evidence suggests that tumors historically classified as cPNET may represent a biologically heterogeneous group of neoplasms, and some cases show molecular similarities to embryonic neuroectodermal tumors (ENT) or other CNS-like tumors (4, 5). These findings highlight the evolving understanding of this rare entity and underscore the importance of integrated morphological, immunohistochemical, and molecular assessments in current diagnostic practices.

PNET occurring in the female reproductive system is extremely rare, with the ovaries being the most common site. Primary tumors of the uterine body with neuroectodermal differentiation are even more uncommon (6). To date, only a limited number of uterine tumors, historically classified as cPNET, have been reported in the literature (**Table 1**), including several small series and case reports (2). Owing to the lack of specificity in clinical presentation, imaging findings, and histopathological features and their overlap with uterine leiomyosarcoma, sex cord-stromal tumors (SCST), and neuroendocrine carcinoma, diagnosis remains highly challenging. Furthermore, the traditional classification of cPNET and pPNET is increasingly being recognized as insufficient for capturing the biological heterogeneity of these tumors.

**Table 1:** Clinical characteristics of the reported cases of cPNET.

Author	Case	Age (years)	Clinical presentation	FIGO stage	Therapy	Diagnosis	Other tumor	Outcome
Nola et al.(2018) (23)	1	39	BP	NA	S, C	cPNET		NED, 24 mo
Chiang et al. (2017) (1)	2	66	AVB	III	S	cPNET Me		DOD, 6 mo
	3	51	AVB	III	S, C	cPNET Mb	EEC	NED
	4	50	AVB	III	S	cPNET Mb	EEC	NA
	5	31	AVB	III	S	cPNET Me	CS	NA
	6	68	NA	IV	S	cPNET Mb		DOD, 12 mo
	7	64	NA	III	S, C	cPNET Mb		NED
	8	NA	NA	NA	NA	cPNET Mb		NA
Elizalde et al. (2016) (14)	9	60	AVB, AP	IV	S, C	cPNET		DOD, 7 mo
Novo et al. (2015) (6)	10	26	AVB, PM	IV	S, C	cPNET		NED, 48 mo
Shimada et al. (2014) (24)	11	63	Constipa- tion	III	S, C	cPNET	NB	NED, 24 mo
Bartosch et al. (2011) (22)	12	58	AVB	IV	S, C	cPNET	EEC	DOD, 11 mo
Dundr et al. (2010) (11)	13	63	AVB	IIIc	S, C	cPNET	RMS	DOD, 7 mo
	14	80	AP	Ib	S, R	cPNET	EC	AWD, 6 mo
	15	79	AVB	Ib	S	cPNET	EC	NED, 29 mo
	16	78	AVB	IIIa	S	cPNET		NED, 8 mo
Euscher et al. (2008) (2)	17	58	AVB, PM	IIIc	S	cPNET		DOD, 2 mo
	18	31	BP	IV	C, R	cPNET		DOD, 20 mo
	19	72	AVB	Ia	NA	cPNET		DOD, 11 mo
	20	48	NA	IIIc	NA	cPNET	EC	NA
	21	81	AVB	NA	NA	cPNET		NA
	22	66	PM	IIIc	S	cPNET	HUC	NED, 41 mo
	23	53	AVB	NA	NA	cPNET		DOD, 22 mo
	24	51	AVB	NA	NA	cPNET		DOD, 12 mo
	25	31	AVB	NA	NA	cPNET		DOD, 26 mo
	26	64	NA	IIb	C, R	cPNET		DOD, 36 mo
	27	64	AVB	NA	NA	cPNET	AS	NA
	28	69	AVB	IV	NA	cPNET		NA
	29	62	NA	IIIc	NA	cPNET	RMS	DOD, 22 mo
	30	55	AVB	Ib	C	cPNET		DOD, 38 mo
	31	52	NA	IV	NA	cPNET		DOD, 6 mo
	32	58	NA	IV	C	cPNET	MMMR	DOD, 6 mo
	33	57	NA	IIIc	C	cPNET	MMMR	DOD, 35 mo
This case	34	46	AP	IIIc2	S, C	cPNET		NED, 24 mo

AP, abdominal pain; AS, adenosarcoma; AVB, abnormal vaginal bleeding; BP, back pain; C, chemotherapy; cPNET, central-type primitive neuroectodermal tumor; CS, carcinosarcoma; DOD, died of disease; EEC, endometrioid endometrial carcinoma; Mb, medulloblastoma; Me, medulloepithelioma; MMMR, malignant mixed Müllerian tumor; mo, months; NA, not available; NB, neuroblastoma; NED, no evidence of disease; PM, palpable mass; R, radiotherapy; RMS, rhabdomyosarcoma; S, surgery. FIGO stages were retained as reported in the original publications. Because the FIGO staging criteria have been revised over time, historical cases were not retrospectively restaged according to the current FIGO 2023 system.

Currently, no standardized treatment protocol is available for these neoplasms. Multimodal strategies, including surgery and combination chemotherapy, which are often based on Ewing’s sarcoma or neuroblastoma regimens, are commonly used, although the overall prognosis remains poor (7). With the increasing application of molecular pathology techniques, particularly next-generation sequencing (NGS) and epigenetic profiling, the molecular characteristics of these tumors are gradually being elucidated, offering new avenues for more precise diagnosis and potentially personalized therapeutic approaches.

We report a rare case of a cPNET arising in the uterine corpus. Clinical, pathological, and molecular features were analyzed. Given the rarity of this tumor and the evolving classification of neoplasms with neuroectodermal differentiation, we provide a comprehensive review of the literature, with particular emphasis on the diagnostic challenges and differential diagnosis of uterine cPNET. This report aimed to improve the recognition of this uncommon entity and contribute to the optimization of its diagnosis and clinical management.

## Case presentation

A 46-year-old female patient presented with persistent left lower abdominal pain. The pain manifested as a continuous dull ache with no identifiable precipitating factors. Menarche occurred at the age of 14 years (gravida 7, para 3), and her last menstrual period occurred 1 week before admission. A comprehensive examination, including pelvic computed tomography (CT), was performed. Pelvic CT scan revealed a rounded soft-tissue mass in the left pelvic cavity with ill-defined boundaries relative to the adjacent left pelvic structures, causing compression of the lower segment of the left ureter. No significant contrast enhancement was observed on the enhanced scan. Concurrently, multiple nodular lesions were visualized within the uterus, exhibiting moderate-to-marked enhancement following contrast administration. Imaging findings suggested a potential pelvic malignant neoplasm accompanied by a uterine leiomyoma (**Supplementary Figures S1A**, **S1B**). Given that the origin of the tumor was unknown and that signs of local invasion were observed, the patient underwent an exploratory laparotomy.

During surgery, a solid mass measuring approximately 9 × 6 cm was observed within the left broad ligament. The mass was relatively hard in texture with a relatively smooth surface, but the boundary with the surrounding tissues was not clear. It was closely attached to and compressed the lower segment of the left ureter. The uterus was irregularly enlarged, and multiple nodular protrusions were visible on the surface. Intraoperative frozen section pathology revealed that the tumor was primarily composed of short spindle-shaped and small round cells with a dense cellular arrangement and notable atypia, but lacked distinctive structural characteristics. A sex cord stromal origin was preliminarily considered, although its malignant potential and definitive tissue origin remain unconfirmed. Because the intraoperative pathological nature and biological behavior of the tumor could not be definitively determined, surgical intervention was restricted to excision of the pelvic mass and local resection of the uterine lesion.

Routine postoperative pathological examination of the pelvic mass revealed a well-circumscribed lesion with an intact capsule and a grey-white to grey-red cut surface with focal cystic degeneration (**Figure 1A**). Histologically, the tumor displayed an infiltrative growth pattern with poorly defined borders (**Figure 1B**) and was composed of diffuse sheets of primitive tumor cells. The neoplastic cells were predominantly small, round to short spindle-shaped, with scant cytoplasm and high nuclear-to-cytoplasmic ratios (**Figure 1C**). Marked nuclear atypia, hyperchromasia, and frequent mitotic figures were observed. In addition, focal rosette-like structures were identified (**Figure 1D**), providing morphological evidence for neuroectodermal differentiation. Notably, the tumor lacked definitive glandular, trabecular, or classic neuroendocrine architectural patterns, and exhibited an overall primitive appearance. Immunohistochemically, the pelvic mass showed diffuse positivity for synaptophysin (Syn) and Insulinoma-associated protein 1 (INSM1), together with CD56 expression and focal perinuclear dot-like staining for chromogranin A (CgA), supporting neuroendocrine/neuroectodermal differentiation (**Figures 1E, F**; **Supplementary Table S1**). The tumor also showed focal Cytokeratin (CK) and CK8/18 expression, indicating partial epithelial differentiation. In contrast, the negative results for CD99 and NK2 homeobox 2 (NKX2.2) argued against ES/pPNET. These findings, together with the primitive morphology and focal rosette-like structures, supported a neuroectodermal neoplasm rather than a conventional epithelial or mesenchymal tumor.

**Figure 1:**
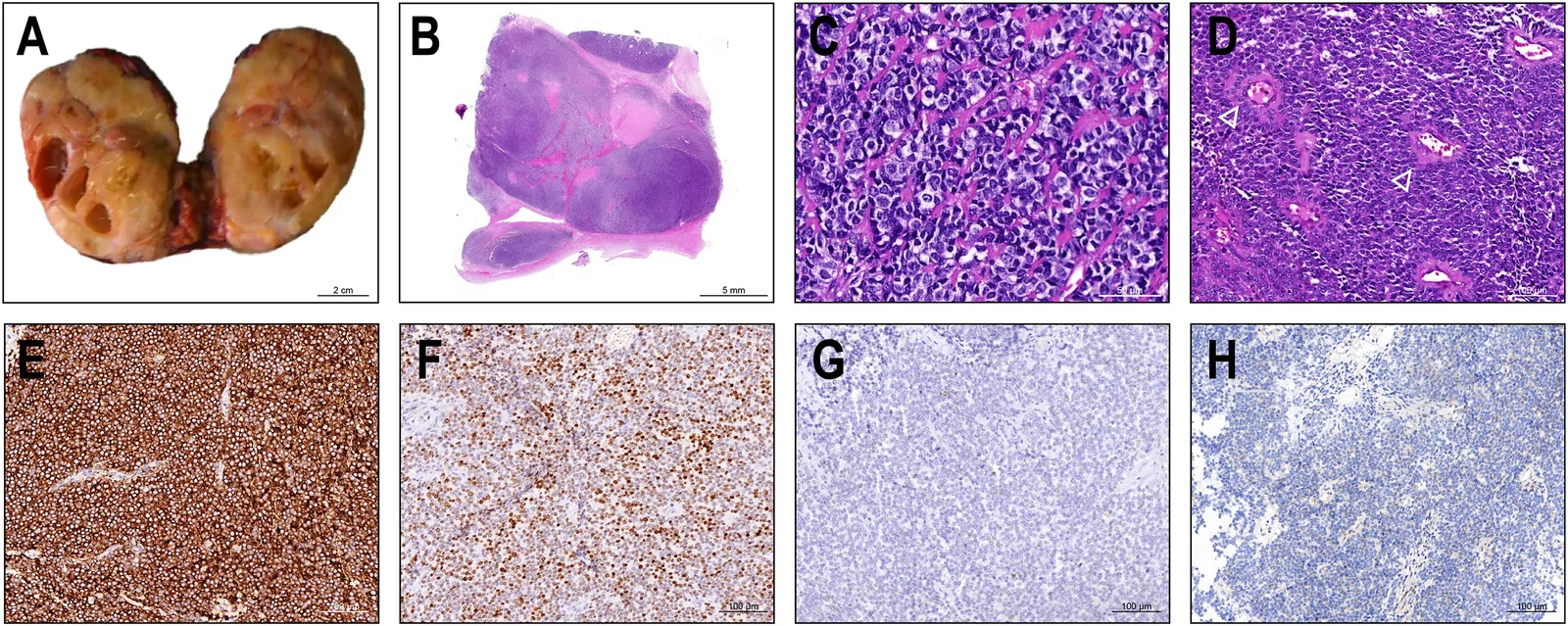
Histopathological and immunohistochemical features of the pelvic mass. **(A)** Gross examination of the resected pelvic mass showing a well-circumscribed lesion with an intact capsule and a grey-white to gray-red cut surface with focal cystic degeneration. **(B)** Hematoxylin and eosin (H&E) staining at very low magnification demonstrates an infiltrative growth pattern of the tumor with ill-defined borders relative to the surrounding tissues (H&E, 1×). **(C)** High-power examination demonstrates densely packed tumor cells with small round to short spindle-shaped morphology and scant stromal background (H&E, 100×). **(D)** Focal rosette-like structures are identified, supporting neuroectodermal differentiation (H&E, 40×; marked with a hollow triangle). **(E)** Tumor cells show diffuse strong positivity for Syn, supporting neuroendocrine differentiation (IHC, 40×). **(F)** INSM1 expression is identified in tumor cells, further supporting neuroectodermal differentiation (IHC, 40×). **(G)** Tumor cells are negative for NKX2.2, arguing against ES/pPNET (IHC, 40×). **(H)** CD99 expression is absent in tumor cells, further excluding ES family tumors (IHC, 40×).

The concurrently resected uterine lesion demonstrated histological features similar to those of the pelvic mass. Microscopically, the tumor showed infiltrative growth within the myometrium with poorly defined margins (**Figures 2A, B**). The neoplasm was composed of diffuse sheets of small, round tumor cells (**Figure 2C**), with some exhibiting vacuolated cytoplasmic changes (**Figure 2D**). Focal collagenous stroma was present, and conspicuous intravascular tumor emboli were identified, indicating aggressive biological behavior. Immunohistochemically, the uterine lesion showed diffuse positivity for Syn and CD56 (**Figures 2E, F**), whereas CK8/18 expression highlighted epithelial-like differentiation (**Figure 2G**). The Ki-67 labeling index reached approximately 40% in hotspot areas (**Figure 2H**), indicating relatively high proliferative activity. Notably, both the pelvic mass and uterine lesion exhibited overlapping epithelial and neuroendocrine immunophenotypic features, while lacking lineage-defining morphological characteristics. The remarkable morphological and immunophenotypic similarities between the two lesions, together with the presence of vascular tumor emboli, suggest a common origin and biologically aggressive behavior. The concurrent expression of both epithelial and neuroendocrine markers indicates a complex admixture of tumor lineages, rendering the primary anatomical origin and biological nature ambiguous. Consequently, a definitive diagnosis could not be established during initial pathological assessment.

**Figure 2:**
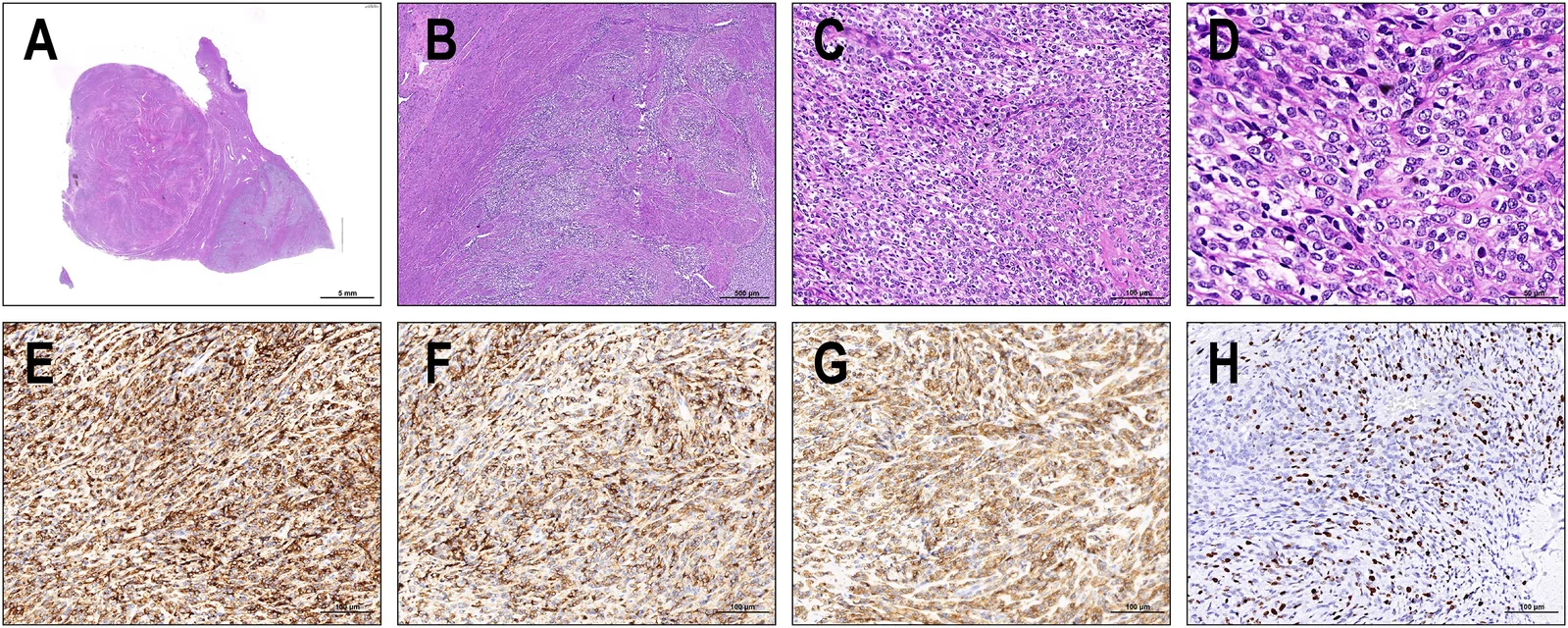
Histopathological and immunohistochemical features of the uterine lesion. **(A)** Hematoxylin and eosin (H&E) staining at very low magnification demonstrates an intramural uterine lesion within the myometrium (H&E, 1×). **(B)** Low-power examination reveals an infiltrative growth pattern with ill-defined borders relative to surrounding tissues (H&E, 10×). **(C)** Intermediate magnification shows diffuse sheet-like proliferation of small round tumor cells (H&E, 40×). **(D)** High-power examination demonstrates small round tumor cells with focal cytoplasmic vacuolated changes (H&E, 100×). **(E)** Tumor cells show diffuse positivity for Syn, supporting neuroectodermal differentiation (IHC, 40×). **(F)** CD56 expression is identified in tumor cells, further supporting neuroendocrine differentiation (IHC, 40×). **(G)** Tumor cells are positive for CK8/18, indicating partial epithelial differentiation (IHC, 40×). **(H)** Ki-67 labeling index is approximately 40% in hotspot areas, indicating relatively high proliferative activity (IHC, 40×).

The patient was subsequently referred to an external institution for pathological consultation. The consultation report concluded that the lesion was malignant, with primary differential diagnoses including high-grade endometrial stromal sarcoma, SCST, and neuroendocrine neoplasms. However, a definitive classification could not be established based on the available morphological and immunohistochemical findings.

To further investigate the molecular characteristics of the tumor and facilitate its subtyping, NGS was performed on the tumor tissue, covering a wide range of genetic mutations, amplifications, and structural variations. These results indicated an intergenic rearrangement between neurofibromatosis type 2 (*NF2*) and BRCA1 A complex subunit 1 (*ABRAXAS1*). Concurrent gene amplification was detected in CRK like proto-oncogene, adaptor protein (*CRKL*), anthrax toxin receptor 2 (*ANTXR2*), C-X-C motif chemokine ligand 13 (*CXCL13*), fibroblast growth factor 5 (*FGF5*), and solute carrier family 34 member 2 (*SLC34A2*). Additionally, single-nucleotide variants (SNV) were identified in TNF receptor superfamily member 14 (*TNFRSF14*), triple functional domain (*TRIO*), ROS proto-oncogene 1, receptor tyrosine kinase(*ROS1*), protein tyrosine phosphatase receptor type O (*PTPRO*), Kruppel like factor 5 (*KLF5*), arachidonate 12-lipoxygenase, 12R type (*ALOX12B*), and Rho associated coiled-coil containing protein kinase 1 (*ROCK1*), none of which exhibited a known definitive classification or established driver significance. Moreover, no specific driver events with clear typing or diagnostic guidance significance or harmful germline mutations were detected (**Supplementary Table S2**). At this point, the NGS findings failed to provide definitive criteria for tumor classification, further highlighting the molecular-level non-specificity and pronounced heterogeneity of this case.

Given the highly invasive nature of the tumor, unclear pathological classification, and risk of potential residual lesions, the patient underwent a multidisciplinary team (MDT) consultation in the gynecologic oncology department. Based on a comprehensive integration of imaging, pathological, and molecular findings, the MDT concluded that the tumor likely originated from the uterus. Extended surgical resection should be performed to ensure adequate tissue sampling for a comprehensive pathological evaluation. Consequently, total hysterectomy, bilateral salpingo-oophorectomy, and pelvic plus para-aortic lymphadenectomy were performed, with omentectomy if necessary.

Ultimately, two months after the initial surgery, the patient was readmitted and underwent the aforementioned definitive surgery. Histopathological examination of the postoperative specimen revealed multiple grayish-white nodular lesions in the myometrium on the left side of the uterus. Review and integration of all surgical specimens revealed densely packed tumor cells arranged in sheet-like and nested patterns, with focal collagenous stromal proliferation and abundant vascular networks. The tumor cells predominantly exhibited a round to short spindle morphology, demonstrating primitive characteristics with inconspicuous nucleoli and brisk mitotic activity. Focal rosette-like structures were observed along with definitive lymphovascular invasion, indicating high malignant potential.

The immunohistochemical findings were further refined in the resected uterine specimen. Tumor cells showed positive staining for Syn, INSM1, and CD56, with perinuclear dot-like positivity for CgA. The Ki-67 proliferation index was approximately 70%, indicating a high-grade neoplasm with prominent neuroendocrine differentiation. Focal positivity for CK8/18 and patchy CK expression further demonstrated partial epithelial differentiation. Negative expression of CD99, Friend leukemia integration 1 (FLI1), and NKX2.2, together with the absence of EWSR1-associated alterations, argued against ES/pPNET. Molecular analysis revealed no EWSR1-associated rearrangement, providing additional evidence against an ES/pPNET-type neoplasm. The absence of definitive glandular, trabecular, or conventional neuroendocrine architecture, together with the primitive cytomorphology and focal rosette-like structures, was considered in the differential diagnosis of high-grade neuroendocrine carcinoma and other small round cell tumors.

The absence of staining for SOX-10, HMB-45, CD3, CD20, CD117, SMA, ER, GATA-3, Caudal-type homeobox 2 (CDX-2), and Villin showed no evidence supporting melanoma, lymphoma, mesenchymal tumors, or other major epithelial mimics. Preserved expression of Integrase interactor 1 (INI-1) and Brahma-related gene 1 (BRG1) excluded tumors associated with SMARCB1/SMARCA4 deficiency. Focal positivity for inhibin-α, scattered Thyroid transcription factor 1 (TTF-1) positivity, and weak PR expression in a small subset of cells were interpreted as nonspecific or ectopic expression. Weak Anaplastic lymphoma kinase (ALK) positivity indicates a potential fusion event, although this was not definitively confirmed using NGS.

Taken together, the diagnosis was not based solely on the exclusion of ES/pPNET. Rather, the diagnosis of uterine cPNET with significant neuroendocrine expression was established through integrated assessment of the tumor’s primitive morphology, focal rosette-like structures, neuroendocrine/neuroectodermal immunophenotype, absence of an EWSR1-associated rearrangement, exclusion of major morphologic and immunophenotypic mimics, and the overall findings across extensively sampled specimens from multiple surgical procedures. The tumor was classified as International Federation of Gynecology and Obstetrics (FIGO) stage IIIC2 according to the current FIGO 2023 staging framework, based on metastatic involvement of the para-aortic lymph nodes below the renal vessels (8). The diagnosis was further reviewed through internal and external pathological consultation and the multidisciplinary team (MDT) discussion.

Postoperatively, the patient received seven cycles of adjuvant combination therapy under the decision of the MDT. The regimen consisted of liposomal doxorubicin and ifosfamide on day 1, cisplatin and ifosfamide on day 2, and cadonilimab and ifosfamide on day 3. After completion of the seven cycles, maintenance therapy with cadonilimab was continued. No specific treatment-related adverse events were documented in the available clinical records, and the patient reported no particular discomfort at follow-up. Follow-up assessments included imaging studies, clinical evaluation, and tumor marker testing. At the 24-month follow-up, the patient remained alive with no evidence of disease (**Figure 3**).

**Figure 3:**
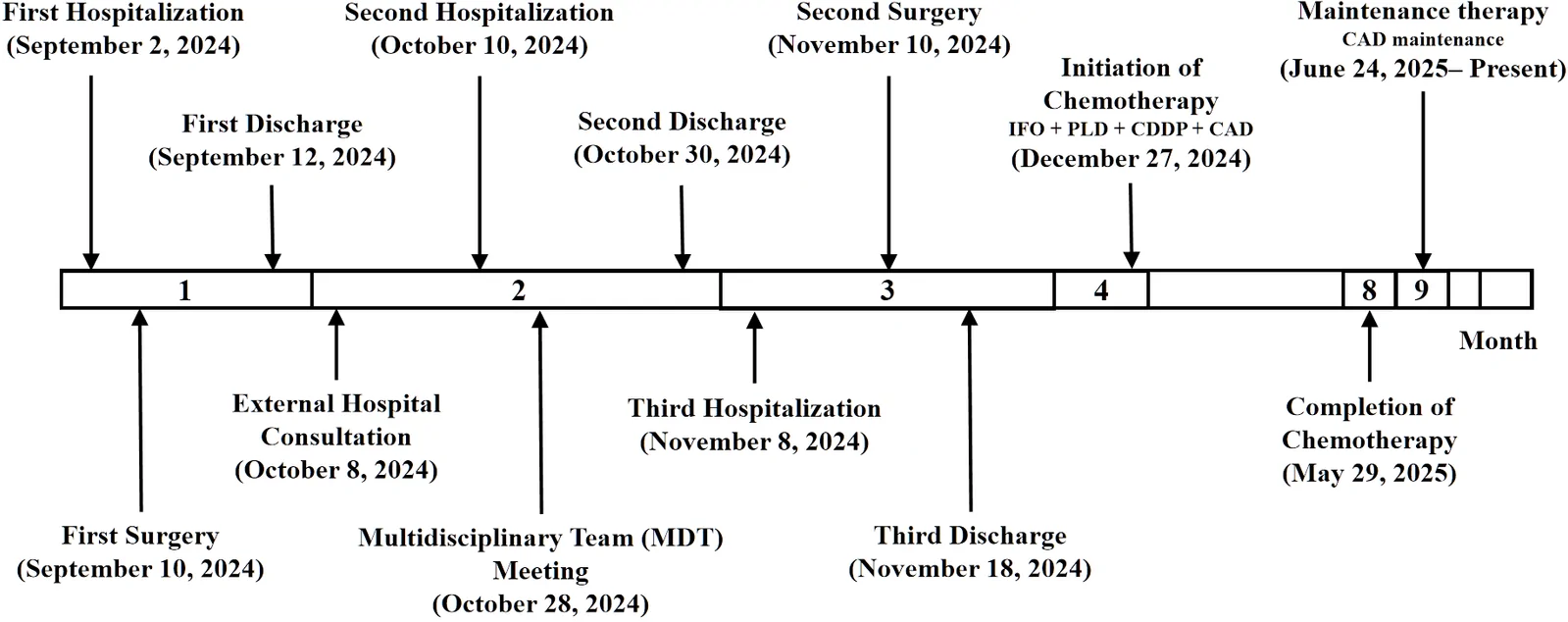
The whole clinical process of this patient. IFO, ifosfamide; PLD, pegylated liposomal doxorubicin; CDDP, cisplatin; CAD, cadonilimab.

## Discussion

PNET was first proposed by Hart and Earle in 1973 and refers primarily to highly malignant undifferentiated small round cell tumors derived from primitive neural tube embryonic cells with the potential for multidirectional differentiation. In 1986, Dehner classified PNET into central (central PNET, cPNET) and peripheral (peripheral PNET, pPNET) types according to the anatomical site of tumor occurrence. Among them, cPNET originates from small round primitive neuroepithelial cells in the supratentorial region and spinal cord, whereas pPNET arises from mesenchymal components derived from the neural crest and is commonly found in the extracranial soft tissue, bone, and peripheral trunk regions. Both pPNET and ES belong to the ES Family Tumors, sharing a significant overlap in histological morphology, immunophenotype, and chromosomal translocations, with pPNET exhibiting more prominent neural differentiation. Since 1993, the WHO has included PNET in the classification system for CNS tumors, and in 2002, it was further grouped under neuroectodermal tumors. Primary uterine cPNETs are rare. Currently,approximately eight articles in the literature reporting over 30 cases of uterine corpus cPNET (**Table 1**), including a large-scale cohort study of 17 cases, are available (2). Its rarity has resulted in a lack of consensus regarding its clinical understanding, diagnosis, and treatment strategies.

However, the origins of uterine PNET remain unclear. The proposed theories include implantation of embryonic CNS tissue after abortion, neuroectodermal metaplasia, unidirectional differentiation of malignant Müllerian mixed tumors, and migration of neural crest cells during embryonic development (9, 10). In uterine tumors with neuroectodermal components associated with other tumor types, the neuroectodermal component may represent a heterologous differentiation pattern in malignant mixed Müllerian tumors, whereas pure uterine neuroectodermal tumors may represent monophasic differentiation of malignant mixed Müllerian tumors (11).

The age of onset of primary uterine PNET shows a bimodal distribution, occurring mainly during adolescence and menopause (12), with cases also reported during pregnancy or the puerperium (13). The clinical manifestations primarily include irregular vaginal bleeding, postmenopausal bleeding, lower abdominal pain, and a palpable pelvic mass. On imaging, it is often misdiagnosed as a uterine fibroid or endometrial carcinoma. Grossly, the tumor often appears multinodular or lobulated with an ill-defined capsule but relatively clear boundaries, which are typically soft, fish-flesh-like in appearance, and accompanied by hemorrhage and necrosis. Macroscopically, the tumor is multinodular or lobulated with relatively well-defined boundaries and an indistinct capsule. Histologically, the tumor consists of relatively uniform, small, round cells arranged in solid sheets or nests, with large areas of necrosis. The nuclei were round or oval, with fine chromatin and inconspicuous nucleoli; mitotic figures were frequently observed, and vascular invasion and perineural infiltration were common. Some of these tumors may express evidence of neural differentiation, such as typical Homer-Wright- or Flexner-Wintersteiner-type rosettes (11).

The definitive diagnosis of uterine cPNET is highly challenging. Excluding other small round cell tumors, such as small cell carcinoma, poorly differentiated carcinoma, endometrial stromal sarcoma, malignant lymphoma, and leukemia (9), and making comprehensive judgments based on morphology, immunohistochemistry, and molecular genetics is necessary. CD99 is one of the most commonly used markers (14), and its strong membranous positivity is highly sensitive for pPNET diagnosis. However, membranous expression of CD99 is not specific and can be found in synovial sarcoma, lymphoblastic lymphoma, and endometrioid adenocarcinoma (15). Therefore, CD99 should be regarded as a supportive rather than a diagnostic (11). The combined detection of neural-related markers, such as FLI1, Vimentin, Syn, and INSM1, can further improve specificity.

At the molecular level, pPNETs/ES commonly harbor characteristic chromosomal rearrangements, most frequently involving EWSR1, whereas such alterations are generally absent in the central type of uterine neuroectodermal tumors reported in the literature. Approximately 85% of pPNETs exhibit t(11;22)(q24;q12) translocation, resulting in the EWSR1-FLI1 fusion gene; 10–15% exhibit t(21;22)(q22;q12) translocation, forming the EWSR1-ERG fusion, and other EWS gene rearrangements can also be observed (16). Of the 17 cases of uterine PNET reported by Euscher et al., 12 underwent FISH testing, and none showed EWSR1 gene rearrangement (2). These findings support the value of molecular testing in distinguishing ES/pPNET from other uterine tumors with neuroectodermal differentiation.

However, the absence of an EWSR1 rearrangement should not, by itself, establish a diagnosis of central PNET. Uterine tumors with primitive morphology and neuroectodermal or neuroendocrine differentiation may show substantial biological and phenotypic heterogeneity, and the differential diagnosis includes high-grade neuroendocrine carcinoma as well as other small round cell neoplasms. In the present case, the diagnosis was therefore based on an integrated evaluation rather than on exclusion of ES alone. The tumor showed a predominantly primitive small round to short spindle cell morphology, focal rosette-like structures, and diffuse expression of Syn and INSM1 with additional CD56 and focal CgA expression, supporting neuroectodermal/neuroendocrine differentiation. At the same time, the absence of CD99, FLI1, and NKX2.2 expression and the lack of an EWSR1-associated rearrangement argued against ES/pPNET. Notably, the tumor lacked definitive glandular, trabecular, or conventional neuroendocrine architecture and retained a predominantly primitive morphology, which was considered in distinguishing it from high-grade neuroendocrine carcinoma. The final diagnosis of uterine cPNET was reached after comprehensive examination of specimens obtained from multiple surgical procedures; integration of morphologic, immunohistochemical, and molecular findings; and internal and external pathological consultation.

Accordingly, the terminology of uterine cPNET should be interpreted cautiously, particularly given the rarity of these tumors and the absence of universally established diagnostic criteria. The present case illustrates the importance of integrating positive morphologic and immunophenotypic evidence with molecular findings and systematic exclusion of major mimics, rather than relying on a single immunohistochemical marker or molecular alteration. Further accumulation of molecularly characterized uterine cases will be necessary to clarify whether tumors currently classified as cPNET represent a biologically homogeneous entity or a heterogeneous group of primitive neoplasms with neuroectodermal differentiation.

NGS revealed structural rearrangements of *NF2*, *ABRAXAS1*, and other genes in adjacent non-coding regions, suggesting the presence of an atypical molecular mechanism. Chromosomal rearrangements and widespread genomic mutations are hallmarks of cancer (17). When breakpoints occur within intronic regions or at exon boundaries, intact fusion transcripts may be preserved. However, if the breakpoints are located within exons, the corresponding transcripts may be disrupted, resulting in altered gene expression profiles. *NF2* is a gene located on the long arm of chromosome 22 (22q) that encodes the Merlin (moezin-ezrin-radixin-like) protein, which belongs to the protein 4.1 family of cytoskeletal elements. Activated Merlin inhibits several signaling pathways that are critical for cell proliferation, including PIKE-L/PI3K, mTORC1, Src/Fak, Mst1/2, Ras/Rac/PAK, ERK1/2, AKT, and CRL4-DCAF. Through these mechanisms, *NF2* functions as a key tumor suppressor, inhibiting the development of various types of cancer (18). Mutations in *NF2* have been associated with more than 11 different types of cancers, including CNS, solitary organ, and skin cancers (19). The Hippo signaling cascade is believed to play a major role in this context. Studies have reported that *NF2* mutations in schwannomas reduce the regulation of the Hippo pathway, promote cell proliferation, and contribute to the pathogenesis of sporadic schwannomas (20). Studies on pediatric brain tumors have also reported that somatic structural variations affect tumor biology by disrupting the *NF2*/Hippo axis (21). *ABRAXAS1* encodes a key subunit of the BRCA1 complex and is involved in homologous recombination repair and DNA damage response (DDR) signaling pathways. Structural alterations in *ABRAXAS1* may lead to dysfunction of the BRCA1 complex, further exacerbating genomic instability, and possibly cooperating with the NF2 pathway for tumor promotion. Thus, uterine cPNET may represent a rare tumor subtype with unique molecular features, possibly involving the cross-regulatory network of the DDR–Hippo pathway, which requires further validation.

Owing to the extreme rarity of uterine cPNETs, no standardized systemic treatment strategy has been established. Most reported cases are treated primarily with surgery, including hysterectomy and bilateral salpingo-oophorectomy, which remains the mainstay of treatment (12). Both chemotherapy and radiotherapy are adjuvant therapies. Various chemotherapy regimens have been reported for uterine PNETs, most of which are based on the protocols for osseous PNETs (22). In the present case, postoperative systemic therapy was determined through MDT discussion. The patient received seven cycles of combination treatment with ifosfamide, liposomal doxorubicin, cisplatin, and cadonilimab, followed by maintenance cadonilimab. Given the absence of an established treatment standard for uterine cPNET, this regimen should be regarded as an individualized treatment strategy rather than as a standardized therapeutic approach for this tumor type. No specific treatment-related adverse events were documented in the available clinical records, and the patient reported no particular discomfort at follow-up. At the 24-month follow-up, the patient remained alive with no evidence of disease. As a single-case report, the present findings cannot be generalized to all patients with uterine cPNET, and the 24-month follow-up remains insufficient to assess long-term recurrence risk and prognosis. Further accumulation of cases and clinical follow-up data will be necessary to determine the efficacy and safety of systemic treatment strategies for uterine cPNET. However, the overall prognosis of patients with cPNET is poor. The relationship between molecular subtypes and prognosis in cPNET warrants further investigation. Additionally, multicenter diagnostic and treatment consensus studies and retrospective cohort studies on uterine cPNET are needed. The therapeutic relevance of *NF2* and *ABRAXAS1* requires further investigation.

## Conclusion

Uterine cPNET is a rare and highly aggressive neoplasm with no established diagnostic or therapeutic standards. An accurate diagnosis requires the integration of histopathological, immunohistochemical, and molecular findings. Given its aggressive clinical behavior and poor prognosis, early recognition and multidisciplinary management are essential. The accumulation of additional cases and molecular data is crucial for improving diagnostic accuracy and developing personalized treatment strategies.

## Data Availability

The original contributions presented in the study are included in the article/**Supplementary Material**. Further inquiries can be directed to the corresponding author.
