# Supplementary material for: Central-type primitive neuroectodermal tumor of the uterus: Case report and literature review
Source: Front Oncol. 2026 Oct 23;16:1926715. doi: 10.3389/fonc.2026.1926715 (PMC13739587; doi:10.3389/fonc.2026.1926715)
Supplement: Supplementary file 1 [file Table1.docx]

**Supplementary Figure 1.** Imaging findings in this case: (A) CT scan reveals a roughly circular area of mixed density in the left pelvic region, measuring approximately 66mm × 61mm (marked with a hollow triangle); multiple nodular soft tissue density shadows are visible within the uterine parenchyma (marked with asterisks). (B) Enhanced scanning reveals an irregularly enhanced pelvic mass and moderate to marked enhancement of uterine nodules. (C) Pelvic CT images following the first surgery. (D) Follow-up pelvic MRI shows no evidence of tumor recurrence.
