# Supplementary material for: Central-type primitive neuroectodermal tumor of the uterus: Case report and literature review
Source: Front Oncol. 2026 Oct 23;16:1926715. doi: 10.3389/fonc.2026.1926715 (PMC13739587; doi:10.3389/fonc.2026.1926715)
Supplement: Supplementary file 2 [file Table2.docx]

**Supplementary Table 1.** Antibodies Used in This Case.

| **Antibodies** | **Full name** | **Clone** | **Location** | **The pelvic mass** | **The uterine lesions** |
| --- | --- | --- | --- | --- | --- |
| ALK | Anaplastic lymphoma kinase | MX064 | C | Weak positive | - |
| BRG1 | Brahma-related gene 1 | MXR028 | N | P | - |
| CD10 | Cluster of differentiation 10 | MX002 | M, C | P | P |
| CD117 | Cluster of differentiation 117 (KIT) | YP145 | M, C | N | - |
| CD20 | Cluster of differentiation 20 | MX003 | M | N | - |
| CD3 | Cluster of differentiation 3 | MX036 | M | N | - |
| CD31 | Cluster of differentiation 31 (platelet endothelial cell adhesion molecule 1, PECAM-1) | MX032 | M | - | Tumor emboli in vessels |
| CD34 | Cluster of differentiation 34 | QBEnd/10 | M, C | N |  |
| CD56 | Cluster of differentiation 56 (neural cell adhesion molecule, NCAM) | MX039 | M | P | P |
| CD99 | Cluster of differentiation 99 | MX111 | M, C | N | - |
| CDX-2 | Caudal-type homeobox transcription factor 2 | MXR024 | N | N | - |
| CgA | Chromogranin A | MX018 | C | Perinuclear dot-like positive | N |
| CK | Cytokeratin | No clone (polyclonal) | C | Partialpositive | N |
| CK8/18 | Cytokeratin 8 and 18 | MX004+  MX035 | C | P | Partialpositive |
| Calretinin | Calretinin | MX027 | C | N | P |
| Cyclin D1 | Cyclin D1 | MXR056 | N | N | - |
| D2-40 | Podoplanin | D2-40 | M, C | - | N |
| Desmin | Desmin | MX046 | C | - | N |
| ER | Estrogen receptor | MXR030 | N | N | N |
| FH | Fumarate hydratase | J-13 | C | - | P |
| FLI1 | Friend leukemia integration 1 | G146-22 | C | N | - |
| GATA-3 | GATA-binding protein 3 | EP368 | N | N | - |
| HMB-45 | Human melanoma black 45 | HMB45 | C | N | - |
| Inhibin-α | Inhibin alpha subunit | MX098 | C | Partialpositive | - |
| INI-1 | Integrase interactor 1 (SMARCB1) | MXR068 | N | P | - |
| INSM1 | Insulinoma-associated protein 1 | MXR064 | N | P | - |
| Ki-67 | Ki-67 proliferation marker | MXR002 | N | 70% | 40% (hotspot) |
| NKX2.2 | NK2 homeobox 2 | EP336 | N | N | - |
| P16 | Cyclin-dependent kinase inhibitor 2A (p16^INK4a^) | MX007 | N, C | Partialpositive | - |
| P53 | Tumor protein p53 | MX008 | N, | N | N |
| PR | Progesterone receptor | MXR008 | N | Partialpositive | N |
| SMA | Smooth muscle actin | 1A4 | N | N | - |
| SOX-10 | SRY-box transcription factor 10 | MXR026 | N | N | - |
| Syn | Synaptophysin | MX038 | C | P | P |
| TTF-1 | Thyroid transcription factor 1 | MX011 | N | Partialpositive | - |
| Vimentin | Vimentin | MX034 | C | P | - |
| Villin | Villin | MXR040 | C | N | - |
| 2SC | S-(2-succino)-cysteine | No clone | M, C | - | N |

**Abbreviations:** N, nucleus. C, cytoplasm. M, membrane. P, positive. N, negative. “-” indicates that the marker was not tested or not reported for that site. All antibodies were ready-to-use and purchased from Fuzhou Maixin Biotech Co., Ltd (Fuzhou, Fujian, China).

**Supplementary Table 2.** The NGS results in this Case.

| **Category** | **Genomic Findings** | **Clinical Interpretation** |
| --- | --- | --- |
| Sequencing platform | DNA sequencing of 706 cancer-related genes; RNA sequencing of 649 genes | Broad-panel NGS for tumor classification and therapeutic stratification |
| Pathogenic / potentially significant alterations | *CRKL*, *ANTXR2*, *CXCL13*, *FGF5*, *SLC34A2*, *ABRAXAS1*, *SMARCB1* amplification; *NF2*/intergenic rearrangement, *ABRAXAS1*/intergenic rearrangement | Predominant copy number alterations without lineage-defining driver events |
| Gene fusions (RNA level) | None detected | Absence of canonical sarcoma- or PNET-associated fusion genes |
| Tumor mutational burden (TMB) | 4.2 mutations/Mb (TMB-L) | Low tumor mutational burden |
| Microsatellite status | Microsatellite stable (MSS) | Unlikely benefit from immune checkpoint inhibitors |
| HLA-I genotype | Heterozygous | Preserved antigen presentation capacity |
| HED score | 7.206 (HED-L) | Low intratumoral immune heterogeneity |
| Germline variants | No pathogenic germline mutations detected | No evidence of hereditary cancer syndrome |
| Actionable alterations | No established targetable alterations identified | Limited role for molecularly targeted therapy |
